# CHANTER syndrome in the context of pain medication: a case report

**DOI:** 10.1186/s12883-024-03748-3

**Published:** 2024-07-22

**Authors:** Nils Jansen, Leonard Wulff, Moritz Conty, Wolf-Rüdiger Schäbitz, Randolf Klingebiel

**Affiliations:** 1 Department of Neuroradiology, Ev. Klinikum Bethel, University Hospital OWL, Campus Bielefeld-Bethel, Bielefeld, Germany; 2 Department of Neurology, Ev. Klinikum Bethel, University Hospital OWL, Campus Bielefeld- Bethel, Bielefeld, Germany

**Keywords:** CHANTER syndrome, POUNCE syndrome, MRI, Pain, Opioids

## Abstract

**Background:**

CHANTER (Cerebellar Hippocampal and Basal Nuclei Transient Edema with Restricted diffusion) is a recently described syndrome occurring in the context of drug abuse. While clinical findings are rather unspecific (disorientation, unresponsiveness), MR imaging (MRI) discloses a characteristic pattern (restricted diffusion in the basal ganglia and hippocampi, cerebellar oedema and haemorrhage), allowing for timely diagnosis before complications such as cerebellar swelling and herniation do occur. Here we report a case of CHANTER primarily based on imaging findings, as there was no evidence of drug abuse on admission.

**Case presentation:**

A 62-year-old Patient was admitted to our hospital after being unresponsive at home. Prehospital intubation was performed, which limited neurological assessment. Under these circumstances no obvious symptoms could be determined, i.e. pupils were isocoric and responsive, and there were no signs of seizures. While the initial CT scan was unremarkable, the subsequent MRI scan showed a distinct imaging pattern: moderately enhancing areas in the basal ganglia and hippocampi with diffusion restriction, accompanied by cerebellar haemorrhage and oedema (Figs. 1 and 2). A comprehensive clinical and laboratory work-up was performed, including drug screening, spinal tap, Holter ECG, echocardiography and EEG. The only conspicuous anamnestic finding was a chronic pain syndrome whose medication had been supplemented with opioids two months previously. The opioid medication was discontinued, which led to a rapid improvement in the patient’s clinical condition without any further measures. The patient was able to leave the intensive care unit and was discharged 10 days after admission without persistent neurological deficits.

**Conclusion:**

Familiarity with typical MRI patterns of toxic encephalopathy in patients from high-risk groups, such as drug abusers, is crucial in emergency neuroradiology. In the presence of typical MRI findings, CHANTER syndrome should be included in the differential diagnosis, even if there is no history of drug abuse, to avoid delay in diagnosis and treatment.

## Background

CHANTER (Cerebellar Hippocampal and Basal Nuclei Transient Edema with Restricted diffusion) is a recently described syndrome that usually occurs in association with drug abuse. Patients usually show disorientation or are unresponsive after drug ingestion [1–4]. Similar reports have previously been published under various names such as ‘opioid-induced amnestic syndrome’ or simply ‘an unusual amnestic syndrome’. Most cases are related to overdose or toxicity in drug addicts using opioids, some also using cocaine or amphetamines. Other causes have also been suggested, such as diffuse hypoxic injury, also secondary to intoxication related to drug abuse [5–9] (Table 1). Similar observations have been made in paediatric patients treated with opioids, for which the acronym POUNCE syndrome [10] was coined.

**Table 1:** Overview of studies with clinical and imaging evidence of the CHANTER syndrome

	Type	Proposed mechanisms	MRI findings
Bhattacharyya et al. 2017	case series(*n* = 16)	opioid, cocaine, hypoxia, stroke	diffusion restriction in hippocampi, basal ganglia, (cerebellum, cortex)
Zhou et al. 2022	case report	opioid/cocaine abuse	cerebellar swelling, basal ganglia lesions
Barash et al. 2017	case series (*n* = 14)	opioids	hippocampal diffusion restriction
Riskallah Alves et al. 2023	case report	cocaine, fentanyl, alcohol	hippocampal diffusion restriction, basal ganglia lesions, cerebellar swelling
Kim et al. 2020	case report	morphine medication	diffusion restriction: cerebellum, occipital, centrum semiovale; cerebellar hemorrhage
Sheehan et al. 2023	case report	opioid overdose	hippocampal diffusion restriction, cerebellar swelling
Bolouri et al. 2004	case report	hypoxic brain injury	diffusion restriction hippocampus and basal ganglia
Decarvalho et al. 2022	case report	opioid abuse	hippocampal diffusion restriction
Yurtsever et al. 2023	case report	substance abuse (unspecific)	diffusion restriction: hippocampus, cerebellum, basal ganglia
Atac et al. 2023	case report	opioids	diffusion restriction: hippocampus, cerebellum, basal ganglia
Jasne et al. 2019	case series (*n* = 6),	opioid abuse/medication	diffusion restriction: hippocampus, cerebellum, basal ganglia
Taylor et al. 2019	case report	fentanyl overdose	hippocampal diffusion restriction
Jingami et al. 2024	case report	opioid intoxication	delayed leukencephalopathy following CHANTER

The term ‘CHANTER syndrome’ was introduced in 2019 by Jasne et al. [2], who shifted the focus to medical imaging given the rather unspecific clinical presentation.

The main features of MR imaging are a combination of restricted diffusion in the basal ganglia and hippocampi, often bilateral, with accompanying cerebellar oedema and mild haemorrhage [1–3, 11–13, and 14]. This specific presentation usually allows for an exclusion of relevant differential diagnoses in the outlined clinical context, most prominently toxic leukoencephalopathy [15, 16], PRES [17], stroke or hypoxic-ischaemic encephalopathy [18], but also infection or a postictal state. Albeit hypoxic-ischaemic encephalopathy can also affect the hippocampus, it usually shows additional infarcts in other areas with a pronounced metabolic requirement, especially in the cerebral cortex. In addition, the presence of cerebellar oedema or cerebellar haemorrhage is unusual in hypoxaemic encephalopathy.

While the pathophysiological mechanisms are not yet clear, the imaging findings in this clinical setting allow for timely diagnosis and neurosurgical intervention if required for decompression in cases of cerebellar herniation [2, 19]. The prognosis is generally favourable, but cases with a poor outcome have also been reported [19].

In contrast to the commonly affected patient group, we report the occurrence of CHANTER syndrome in a patient, who was chronically treated with pain medication, and did neither have a history of drug abuse nor an apparent overdose on admission.

## Case presentation

A 62-year-old male patient was admitted to our hospital after becoming unresponsive at home. Relatives reported similar episodes of delayed response in the previous week. Prior to admission, the patient had been found unresponsive sitting at the kitchen table at 5 a.m. with no signs of an epileptic seizure. Intubation was required prior to admission, which limited the neurological assessment. Under these circumstances, no obvious symptoms could be detected, i.e. the pupils were isocoric and reactive.

On admission and during subsequent monitoring, the patient showed episodic elevated blood pressure of up to 180/100 mm Hg, which normalised with antihypertensive treatment.

The initial CT scan including CT angiography and CT perfusion was unremarkable, i.e. no signs of ischaemic stroke or cerebral haemorrhage were detected. There was minimal vasoclerosis of the carotid bifurcation without significant stenosis or intracranial vascular occlusion.

The patient was admitted to our intensive care unit and, after extubation, transferred to the stroke ward for further assessment.

Comprehensive clinical and laboratory investigations were performed, including lumbar puncture, Holter ECG, echocardiography and EEG without significant pathological findings. In particular, there were no indications for seizures, cardiac arrhythmias or neural infection detectable.

The only significant anamnestic finding was a chronic pain syndrome, the medication for which had been supplemented with hydromorphone two months previously. The opioid levels measured on the day of admission were within the expected therapeutic range, and the patient also denied any relevant changes in dosage.

**Fig. 1:**
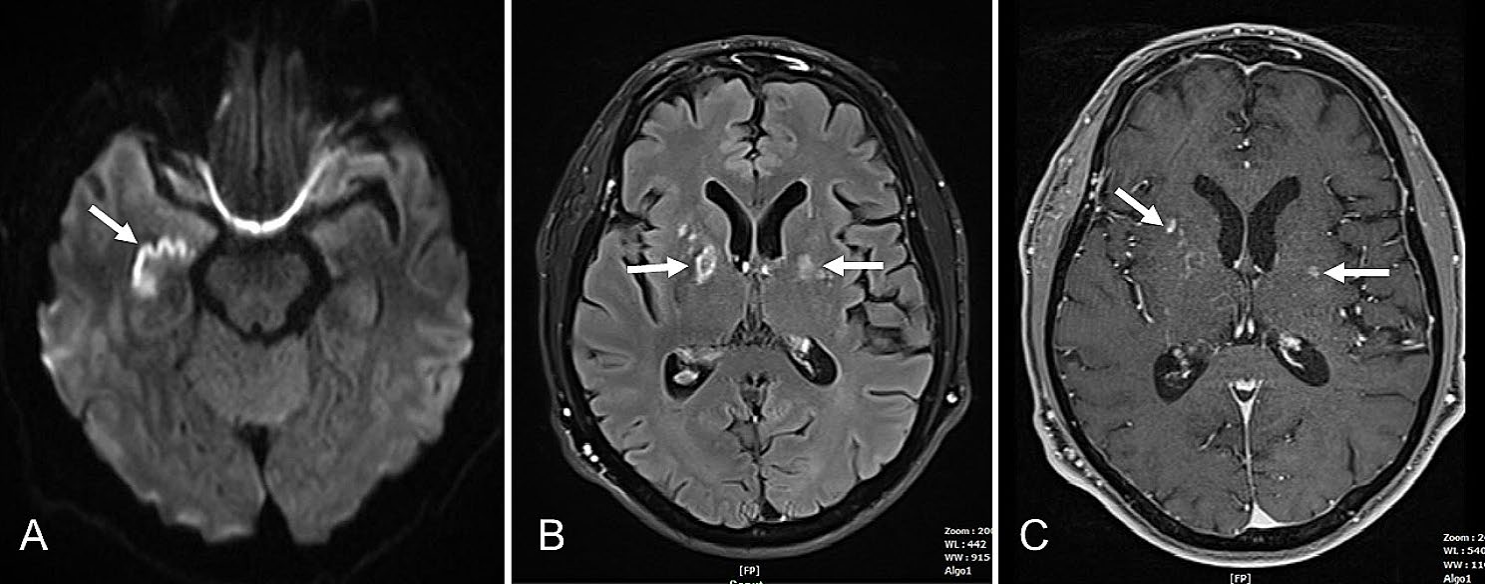
**A-C**: MRI scan on day 6. DWI **(A)**, FLAIR **(B)** and contrast enhanced T1w images **(C)**, disclosing restricted hippocampal diffusion **(A)** and basal ganglia lesions **(B)** with contrast enhancement **(C)**

**Fig. 2:**
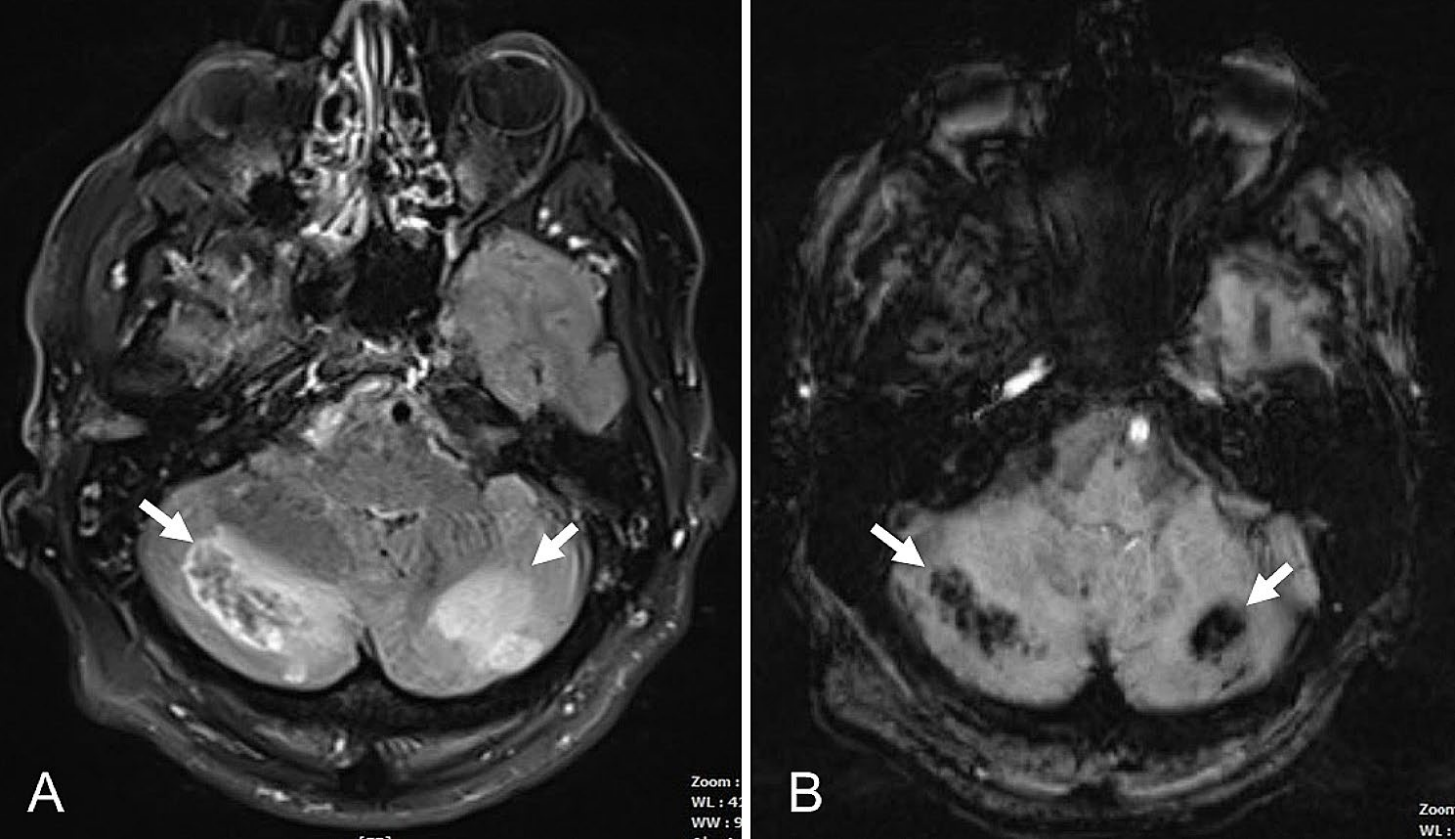
**A-B**: MRI scan on day 6. FLAIR **(A)** and susceptibility weighted **(B)** images, showing bilateral hemorrhagic **(B)** cerebellar lesions, complementing the typical imaging findings of CHANTER syndrome in Fig. 1

The subsequent MRI showed a conspicuous imaging pattern: moderately enhanced areas in the basal ganglia and hippocampi with diffusion restriction, accompanied by cerebellar haemorrhages and oedema (Figs. 1 and 2). Knowing the previously published cases, opioid medication was immediately discontinued under the working diagnosis of CHANTER syndrome, which led to a rapid improvement in the patient’s clinical condition without further intervention.

The differential diagnoses of PRES and ischaemic stroke did not appear to be compatible with the imaging findings and were consecutively excluded.

Hypoxic-ischaemic encephalopathy and carbon monoxide poisoning, which can also affect the hippocampus, tend to show additional infarcts in other areas susceptible to metabolic stress, usually the cerebral cortex. In addition, cerebellar oedema or haemorrhage would be uncommon findings in this context. As the carbon monoxide levels on admission were normal and there were no corresponding clinical features, carbon monoxide poisoning, which can look very similar on MR images, was also ruled out. The cerebellar oedema remained mild on follow-up and there was no risk of tonsillar herniation. The patient was able to leave the ICU and was discharged 10 days after admission with no neurological deficits.

Opioid medication was switched to oxycodone as, unlike hydromorphone, no evidence of associated CHANTER syndrome has been reported to date.

Clinical and MRI follow-up examinations after 6 and 12 months showed further improvement with only minimal residual gliosis of the basal ganglia and haemosiderin deposits in the cerebellar hemispheres.

Apart from a very unspecific occasional dizziness, no neurological symptoms occurred in the further course.

## Discussion

CHANTER (Cerebellar Hippocampal and Basal Nuclei Transient Edema with Restricted Diffusion) has recently been described as a cause of acute disturbance of consciousness in patients, often due to drug abuse in adults [1, 2].

The syndrome has distinct imaging patterns, namely areas of restricted diffusion and enlarging lesions of the basal ganglia and hippocampus, as well as bilateral cerebellar oedema and haemorrhage, as in our case. The combination of these features in the appropriate clinical setting distinguishes it from other differential diagnoses, most notably stroke, PRES, hypoxic-ischaemic encephalopathy, carbon monoxide poisoning, toxic leukoencephalopathy, infections and neoplasms.

Timely diagnosis is crucial to avoid complications such as progressive cerebellar oedema with obstructive hydrocephalus [1, 2, and 19] or tonsillar hernia.

The disease appears to be self-limiting once substance exposure is discontinued [1–3], although severe cases with only limited recovery have also been described. As the symptoms are non-specific and the diagnosis is currently based on the abnormal imaging pattern, further studies are needed to determine the underlying pathophysiological mechanisms.

In contrast to previous cases, where the onset of symptoms was clearly correlated with opioid overdose, normal opioid serum levels were found on admission. Therefore, a non-dose related mechanism should be considered. A delayed course of the disease due to previous intoxication could also be discussed as an alternative [20], even though there was no MRI-based evidence of a polyphasic course in our case.

## Conclusion

We diagnosed CHANTER syndrome in a patient with no obvious affiliation to an established risk group. Patients suffering from this syndrome are difficult to assess based on clinical findings alone and therefore require a high degree of suspicion and timely MR imaging to avoid a critical outcome.

## Data Availability

All data generated or analysed during this study are included in this published article.

## Abbreviations

CHANTER: Cerebellar Hippocampal and Basal Nuclei Transient Edema with Restricted diffusion
CT: Computed tomography
DWI: Diffusion-weighted imaging
ER: Emergency room
FLAIR: Fluid-attenuated inversion recovery
GCS: Glasgow Coma scale
MRI: Magnetic resonance imaging
